# Eu^2+^-Activated Green-Emitting Phosphor Obtained from Eu^3+^ Ions doping Zeolite-3A in Air Surroundings and Its Efficient Green Light-Emitting Diodes

**DOI:** 10.1186/s11671-019-3130-8

**Published:** 2019-08-28

**Authors:** Honge Wu, Guang Tao Fei, Xiao Li Ma, Ze Min Hu, Xu Dong Gao, Yong Shuai Wei, Junxi Zhang, Li De Zhang

**Affiliations:** 1 Key Laboratory of Materials Physics and Anhui Key Laboratory of Nanomaterials and Nanotechnology, Institute of Solid State Physics, Hefei Institutes of Physical Science, Chinese Academy of Sciences, P.O. Box 1129, Hefei, 230031 People’s Republic of China; 2 University of Science and Technology of China, Hefei, 230026 People’s Republic of China; 3 College of Biological and Chemical Engineering, Anhui Polytechnic University, Wuhu, 241000 People’s Republic of China; 4 Department of Opto-electronic Information Science and Engineering, School of Instrument Science and Opto-electronics Engineering, Hefei University of Technology, 193 Tunxi Road, Hefei, 230009 People’s Republic of China

**Keywords:** Zeolite-3A, Divalent europium, Green-emitting phosphor, High-thermal reaction, Green LED

## Abstract

Eu^2+^-activated phosphors are widely applied in lighting and display areas because of their good optical performance. In this paper, an excellent green-emitting zeolite-3A: 1.3 wt% Eu phosphor is prepared by a green and eco-friendly high-thermal reaction method without any reducing atmosphere or agents. Meanwhile, the reducing mechanism from Eu^3+^ ions to Eu^2+^ ions is investigated. The experiment results show that the morphology, crystal structure, and luminescent property are affected by sintering temperature. The resulting sample shows the broad excitation band is in the range of 310–450 nm and the peak of the broad emission band is located at 523 nm. Furthermore, zeolite-3A: 1.3 wt% Eu phosphor is encapsulated on a commercial UV-emitting chip to fabricate a purity green light-emitting diode (LED) with the Commission Internationale de L’Eclairage (CIE) color coordinates at (0.295, 0.537).

## Background

Luminescent materials are widely used in many fields, such as lighting and display devices [1–5]. Over the years, rare-earth (RE) europium (Eu)-activated luminescent materials have received more attention due to their unique optical properties, such as high brightness [6, 7], high chemical stability [8, 9], and excellent eco-friendliness [8, 10]. In particular, Eu ions have two oxidation states of divalent (Eu^2+^) and trivalent (Eu^3+^), exhibiting different emitting characteristics. Generally, Eu^3+^ ions are mainly as a red-emitting activator which originated from ^5^D_0_ → ^7^F_J_ (*J* = 1, 2, 3, 4, and 5) transitions [11–13]. However, Eu^2+^ ions, their 5d electrons located in the outer orbitals, are susceptible to their surroundings. Thus, their emissions are easily affected by the crystal field environment, having a wide region from ultraviolet (UV) to red. Chen et al. prepared Eu^2+^-activated fluorophosphates Ba_3_GdNa(PO_4_)_3_F with blue and red double-color-emitting phosphor [14]. Sato et al. reported red-emitting Ca_2_SiO_4_:Eu^2+^ phosphors [15]. Lin et al. synthesized Eu^2+^, Mn^2+^-activated Ca_9_Mg(PO_4_)_6_F_2_ phosphors with blue to yellow color emission [16]. It is suggested that Eu^2+^-activated phosphors caused by parity-allowed 5d–4f electronic transitions show strong broad emission band [7]. Therefore, the Eu^2+^-activated phosphors are the main focus of luminescent materials in the recent years.

Nowadays, Eu^2+^-doped luminescent materials are obtained by reducing Eu^3+^ to Eu^2+^, because there is no natural Eu^2+^-doped materials. Usually, it can be realized in a reducing atmosphere including H_2_, H_2_/N_2_, or CO. For example, Gao et al. recently obtained Eu^2+^-activated phosphor from Eu^3+^-exchanged USY (Na_28_Si_168_Al_28_·240H_2_O, Si/Al ratio = 6) zeolites by thermal treatment in H_2_/N_2_ reducing atmosphere [17]. Chen et al. reported that Eu^2+^-sensitized Sr_6_Ca_4_ (PO_4_)_6_F_2_:Tb^3+^ phosphor could be obtained by using high-temperature solid-state method in H_2_/N_2_ reducing atmosphere [18]. Nevertheless, the reaction in reductive atmosphere is relatively dangerous and requires well-equipped working environment, which leads to a higher cost of phosphors. In addition, the environmental pollution will be produced if the reaction is proceeded under CO-reducing conditions. Therefore, a green, eco-friendly, and lower cost preparation method has attracted more attention.

As is well known, Eu^3+^ among some special compound hosts, such as borates [19], phosphates [20], and aluminates [21], can also be reduced to Eu^2+^ in air at a high temperature. It is suggested that all these compounds contain rigid tetrahedral BO_4_, PO_4_, AlO_4_, or octahedral AlO_6_ group framework, surrounding and insulating the produced Eu^2+^ ions from oxygen [21, 22]. Zeolites, as one kind of aluminosilicate, not only are natural minerals, but also can be synthesized in industry at a lower cost [23–28]. Notably, their structure can satisfy aforementioned requirements, reducing Eu^3+^ ions to Eu^2+^ ions and making Eu^2+^ ions stable. They are also widely used as excellent host materials for luminescent material applications because of the high chemical stability [29, 30] and so on. Among various zeolites, zeolite-3A ($\frac{2}{3}$K_2_O·$\frac{1}{3}$Na_2_O·Al_2_O_3_·2SiO_2_·$\frac{9}{2}$H_2_O, Si/Al ratio ≈ 1) has been used as host material for down-conversion phosphor. Herein, we achieve Eu^2+^-activated zeolite-3A phosphor through high-thermal treatment method without any reducing atmosphere. The obtained Eu^2+^-activated zeolite-3A phosphor has a quantum yield of about 36.6%. This preparation method is safe, green, and environmentally friendly. The broad excitation band of the sample we obtained is in the range of 310–450 nm, and the peak of emission band is located at 523 nm. And the samples are stable and easily re-prepared. By encapsulating green-emitting zeolite-3A: 1.3 wt% Eu phosphor on a UV-emitting chip, we acquire a good green LED with the Commission Internationale de L’Eclairage (CIE) color coordinates at (0.295, 0.537) and brightness of 231.6 cd/m^2^ under 3 V voltage. These results not only show a simple and eco-friendly preparing approach but provide an excellent green emission phosphor with promising applications in the fields of lighting and display.

## Methods

### The Aim of the Study

We aim to prepare Eu^2+^-activated phosphors with bright green emission through safe, green, and environmentally friendly synthesis method, without any reducing atmosphere.

### Materials

Zeolite-3A ($\frac{2}{3}$K_2_O·$\frac{1}{3}$Na_2_O·Al_2_O_3_·2SiO_2_·$\frac{9}{2}$H_2_O, Si/Al ratio ≈ 1) purchased from Shanghai Tongxing Molecular Sieve Co., LTD, and europium oxide (Eu_2_O_3_) obtained from Sinopharm Co., Ltd, were used without any further purification. Silicone resin and InGaN blue chip (5 mm × 5 mm, *λ* = 375 nm) were received from Shenzhen Looking Long Technology Co., Ltd.

### Synthesis of Samples

Eu^2+^-actived zeolite-3A samples were prepared by using a typical high-temperature solid-state reaction method. Firstly, different stoichiometric amounts of zeolite-3A and Eu_2_O_3_ were mixed well and thoroughly ground in an agate mortar for 40 min. And then, they were sintered at different temperature without reducing atmosphere. Finally, the target samples were gained after cooling.

### Fabrication of Green LEDs

Eu^2+^-actived zeolite-3A powder and silicone resin were blended according to 1:5 of mass ratio, then stirring homogeneously. The composition was coated on an InGaN chip and cured under 60 °C for about 2 h. Finally, the thickness of the composite was measured to be about 1 mm.

### Characterization

The morphology and structure of the resulting products were characterized through field emission scanning electron microscope (FESEM, FEI Sirion-200) and X-ray diffraction (XRD, Philips X’Pert) with Cu Kα radiation (*λ* = 0.15405 nm). The thermogravimetric analysis (TG) curves were measured by SDT Q600 V20.9 Build 20, which were obtained from room temperature to 800 °C with a heating rate of 10 °C/min in a nitrogen atmosphere (flow rate 10 ml/min). The photoluminescence excitation (PLE) and photoluminescence (PL) spectra were obtained at room temperature using Edinburgh Instruments FLS920 Time Resolved and Steady State Fluorescence Spectrometers equipped with a 450-W Xe lamp. The oxidation state of europium element was investigated by X-ray photoelectron spectroscopy (XPS, ESCALAB 250). Electroluminescence (EL) spectrum was investigated by Ocean Optics FLAME-S-VIS-NIR spectrometer with a fiber integrating sphere (FOIS-1) and a Keithley 2400 electrometer.

## Results and Discussion

**Fig. 1:**
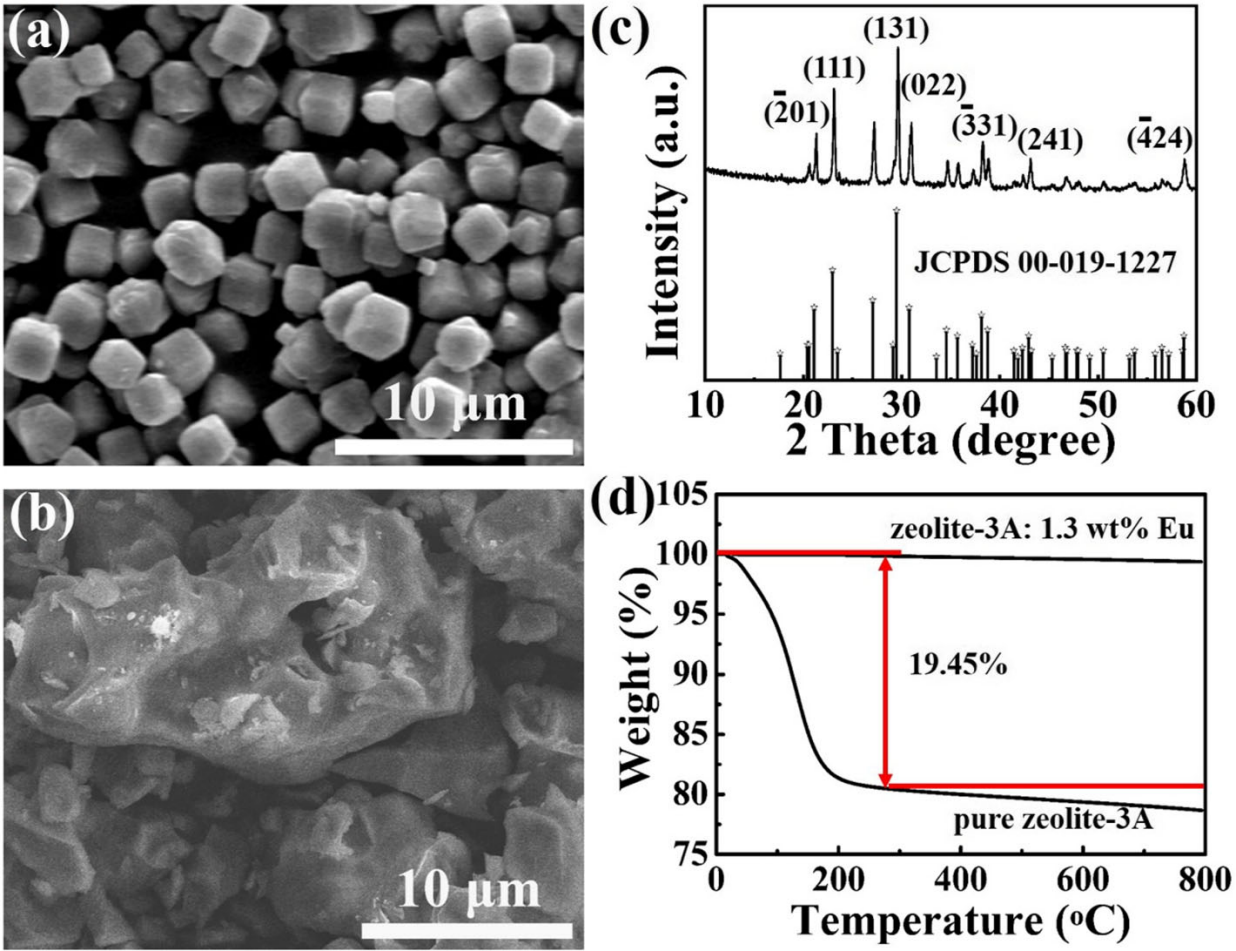
The SEM images of **a** pure zeolite-3A and **b** zeolite-3A: 1.3 wt% Eu phosphor sintered at 1400 °C for 3 h. **c** XRD pattern of zeolite-3A: 1.3 wt% Eu phosphor sintered at 1400 °C for 3 h. **d** TG curves of pure zeolite-3A and zeolite-3A: 1.3 wt% Eu phosphor sintered at 1400 °C for 3 h

Figure 1a shows the SEM image of pristine zeolite-3A. It can be observed that the morphology of the pristine zeolite-3A is irregular cubic structure with the side length of about 1.5 μm. Zeolite-3A: 1.3 wt% Eu phosphors are obtained through high-temperature solid-state reaction method, without any reducing atmosphere. The morphology and structure of zeolite-3A: 1.3 wt% Eu phosphor obtained at 1400 °C for 3 h are characterized by the field emission scanning electron microscope and X-ray diffraction measurements, as shown in Fig. 1b, c, respectively. Figure 1b shows that the particles exhibit irregular morphology structure and the distribution of the crystallite sizes is not uniform. Comparing Fig. 1b with a, it can be found that the particles of zeolite are aggregated with each other after sintering at 1400 °C for 3 h. As can be seen in Fig. 1c, all the diffraction peaks in XRD pattern of zeolite-3A: 1.3 wt% Eu phosphor are in good agreement with the pure zeolite-3A phase (JCPDS no. 00-019-1227), and no other impurity peaks are observed. It means that Eu^2+^ ions are successfully introduced into the zeolite host lattices and a certain amount of Eu^2+^ ions doping do not obviously change the crystal structure [10]. Figure 1d shows TG curves of pure zeolite-3A and zeolite-3A: 1.3 wt% Eu phosphor sintered at 1400 °C. It can be seen that there is a continuous mass loss during heating up to about 266 °C in TG curve for pure zeolite-3A, where it reaches a value about 19.45%. This is corresponded to the liberation of physically bound water localized in the zeolite-3A cavities and channels [31]. The phenomenon of mass loss is not obvious as increasing temperature sequentially. As seen from TG curve of zeolite-3A: 1.3 wt% Eu phosphor sintered at 1400 °C, there is almost no mass loss. These results indicate that zeolite-3A: 1.3 wt% Eu sintered at 1400 °C is very stable.

**Fig. 2:**
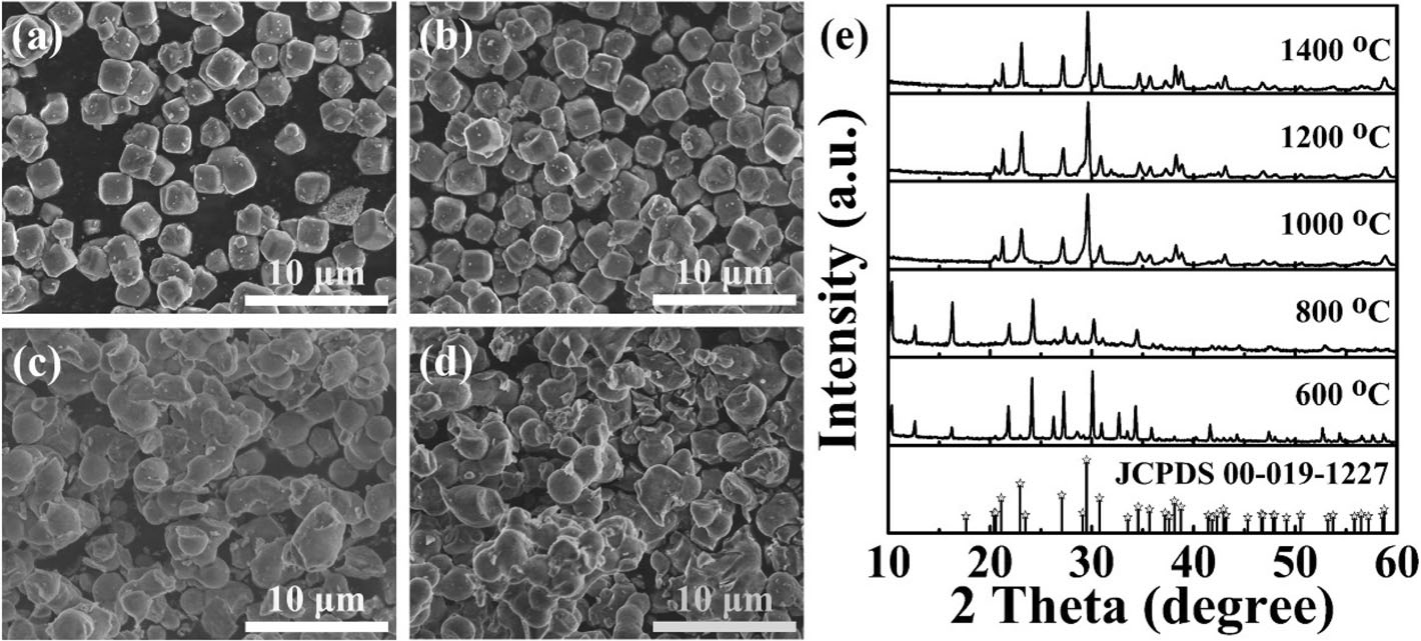
SEM images of zeolite-3A: 1.3 wt% Eu phosphors sintered at 600 °C (**a**), 800 °C (**b**), 1000 °C (**c**), and 1200 °C (**d**), respectively. **e** XRD patterns of zeolite-3A: 1.3 wt% Eu phosphors sintered at different temperatures, respectively

Figure 2 shows the SEM images and XRD patterns of the zeolite-3A: 1.3 wt% Eu phosphors sintered at different temperature. From the SEM images (Fig. 2a–d), it is clear to observe that the morphology changes significantly with the increase of the sintering temperature. When the sintering temperature is relatively lower (600 °C and 800 °C), the morphology of samples still keeps as the pristine zeolite-3A, that is, in cubic shape with an average size of 1.5 μm (shown in Fig. 1a). However, it can be observed that the particles begin to aggregate when the sintering temperature reaches 1000 °C and 1200 °C. As the sintering temperature increases, the particles can continue to aggregate and form massive structure (Fig. 1b). Meanwhile, their XRD patterns are shown in Fig. 2e. It is noticeable that the diffraction peaks of samples prepared at 600 °C and 800 °C are not absolutely indexed to the pure zeolite-3A standard card (JCPDS no. 00-019-1227). Two samples exist extra diffraction peaks located at 12.5° and 16.3°, which are assigned to the peaks of Eu_2_O_3_ (JCPDS no. 00-012-0384). It means that Eu ions cannot be successfully incorporated in the zeolite host lattices when the calcination temperature is lower than 800 °C [32]. Nevertheless, the samples sintered at above 1000 °C show peaks corresponding to the major characteristic peaks of pure zeolite-3A standard card.

**Fig. 3:**
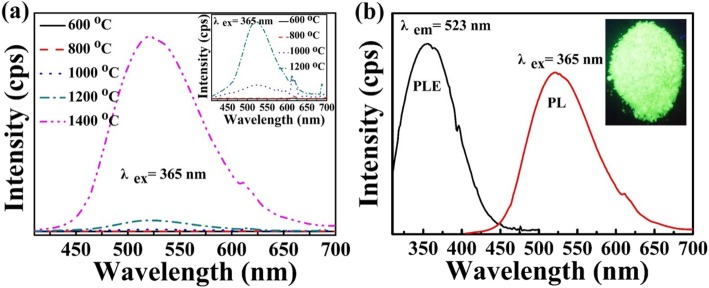
**a** PL emission spectra for zeolite-3A: 1.3 wt% Eu phosphors sintered at different temperatures, respectively. The inset shows the magnifying spectra. **b** photoluminescence excitation (PLE) and photoluminescence (PL) emission spectra for zeolite-3A: 1.3 wt% Eu phosphors sintered at 1400 °C for 3 h. The inset is the photograph of the sample with the illuminaton of a 365-nm UV lamp

To investigate the effect of calcination temperature on PL emission, the PL emission spectra of the samples at different calcination temperatures are tested and shown in Fig. 3a. As seen in the inset, the samples sintered at 600 °C and 800 °C only show the red emission peak centered at 617 nm which is attributed to the ^5^D_0_ → ^7^F_2_ electric-dipole transition of Eu^3+^ ion [33]. As the sintering temperature increases, the red emission peak becomes weak gradually and the green emission peak (centered at 523 nm) is obviously observed. Especially, the sample prepared at 1400 °C mainly shows a typical characteristic Eu^2+^ emission centered at 523 nm, which is due to the 4f^6^5d → 4f^7^ transition [34]. By comparing the different PL curves in Fig. 3a, it can be observed that the amount of Eu^3+^ → Eu^2+^ continuously increases with increasing sintered temperature. This result coincides with that of the XRD patterns in Fig. 2e. Namely, Eu^3+^ ion is the main form when the calcination temperature is lower than 1000 °C. Eu^3+^ ions are gradually reduced to Eu^2+^ ions when the sintering temperature is higher than 1000 °C. Figure 3b shows the PLE and PL spectra of the sample sintered at 1400 °C. It can be found that the PLE spectrum shows a broad excitation band between about 310 and 450 nm [7, 35]. And the inset is the photograph showing a bright green light emission with the illumination of a 365-nm UV lamp. The PL spectrum in Fig. 3b is consistent with the color witnessed in the photograph.

**Fig. 4:**
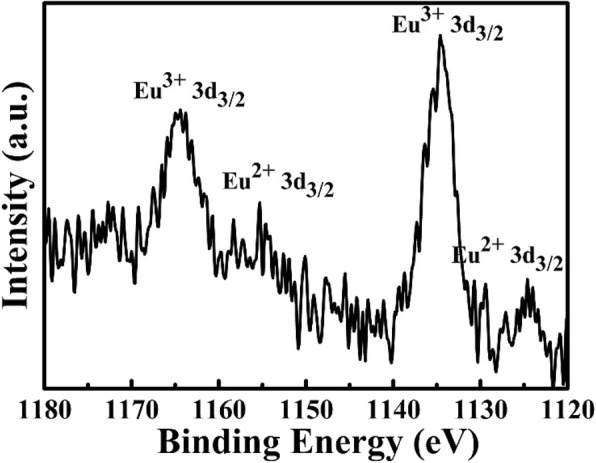
XPS spectrum for Eu element of the zeolite-3A: 1.3 wt% Eu phosphors sintered at 1400 °C for 3 h

In order to further investigate the oxidation state of Eu element, the XPS pattern of Eu3d for the sample obtained at 1400 °C is monitored and shown in Fig. 4. The peaks located at 1165 eV and 1135 eV are corresponded to Eu^3+^ oxidation state, but the peaks around 1155 eV and 1125 eV are attributed to Eu^2+^ oxidation state [11, 36]. This result shows that some of the Eu^3+^ ions are reduced to Eu^2+^ ions in the zeolite host under high-thermal treatment reaction, and this result is consistent with the PL spectra (Fig. 3). The possible reaction mechanism may be shown in the following equations:

1 $$ {\mathrm{Eu}}_2{\mathrm{O}}_3\overset{{\mathrm{K}}_2\mathrm{O}}{\to}\kern0.5em 2{\left[{\mathrm{Eu}}^{3+}\right]}_{\mathrm{K}}^{\ast \ast }+\kern0.5em 4{V}_{\mathrm{K}}^{\prime }+3{\mathrm{O}}_{\mathrm{O}}^{\times } $$

2 $$ {V}_{\mathrm{K}}^{\prime}\to {V}_{\mathrm{K}}^{\times}\kern0.5em +{\mathrm{e}}^{\prime } $$

3 $$ {\left[{\mathrm{Eu}}^{3+}\right]}_{\mathrm{K}}^{\ast \ast}\kern0.5em +{\mathrm{e}}^{\prime}\to {\left[{\mathrm{Eu}}^{2+}\right]}_{\mathrm{K}}^{\ast } $$

4 $$ {\left[{\mathrm{Eu}}^{2+}\right]}_{\mathrm{K}}^{\ast }+{\mathrm{e}}^{\prime}\to {\left[{\mathrm{Eu}}^{2+}\right]}_{\mathrm{K}}^{\times } $$

Here, [Eu^3+^]_K_ and [Eu^2+^]_K_ represent Eu^3+^ and Eu^2+^ ions substitution for K^+^ ions position, respectively; O_o_ represents the position of an oxygen for oxygen in the matrix crystal; and V_K_ is the vacancy of K^+^ ion. Superscript “*,” “**,” “′,” and “×” indicate one positive charge, two positive charges, one negative charge, and electroneutrality, respectively. During sintering, Eu^3+^ ion replaces K^+^ position in the zeolite. To keep the charge balance, one Eu^3+^ ion will substitute for three K^+^ ions. Thus, two vacancy defects of K^+^ ions (*V*_K_^′^) and one defect of Eu^3+^ ion ([Eu^3+^]_K_^**^), which carries one negative charge and two positive charges in appearance, respectively, will create in zeolite host (seen from Eq. (1)). Then, the vacancy *V*_K_^′^ will act as a donor of electrons (seen from Eq. (2)), and the [Eu^3+^]_K_^**^ defect is the acceptor of the electrons. At high temperature, [Eu^3+^]_K_^**^ will capture one electron from the vacancy of K^+^ ions (*V*_K_^′^) and this electron will be filled into the 4f orbit of Eu ion. Therefore, Eu^3+^ ion is reduced to Eu^2+^ and [Eu^3+^]_K_^**^ defect becomes [Eu^2+^]_K_^*^ defect (seen from Eq. (3)). At this time, the position of Eu^2+^ ion is with apparent one positive charge. [Eu^2+^]_K_^*^ defect would attract the negative electron of another K^+^ vacancy to the surrounding of itself and becomes apparent electroneutrality [Eu^2+^]_K_^×^ (shown in Eq. (4)) [11, 21, 22, 37–41].

It is suggested that the rigid three-dimensional tetragonal framework of AlO_4_ and SiO_4_ can surround Eu^2+^ ions and insulate them from oxygen, and then, Eu^2+^ can exist steadily in our aim phosphors.

**Fig. 5:**
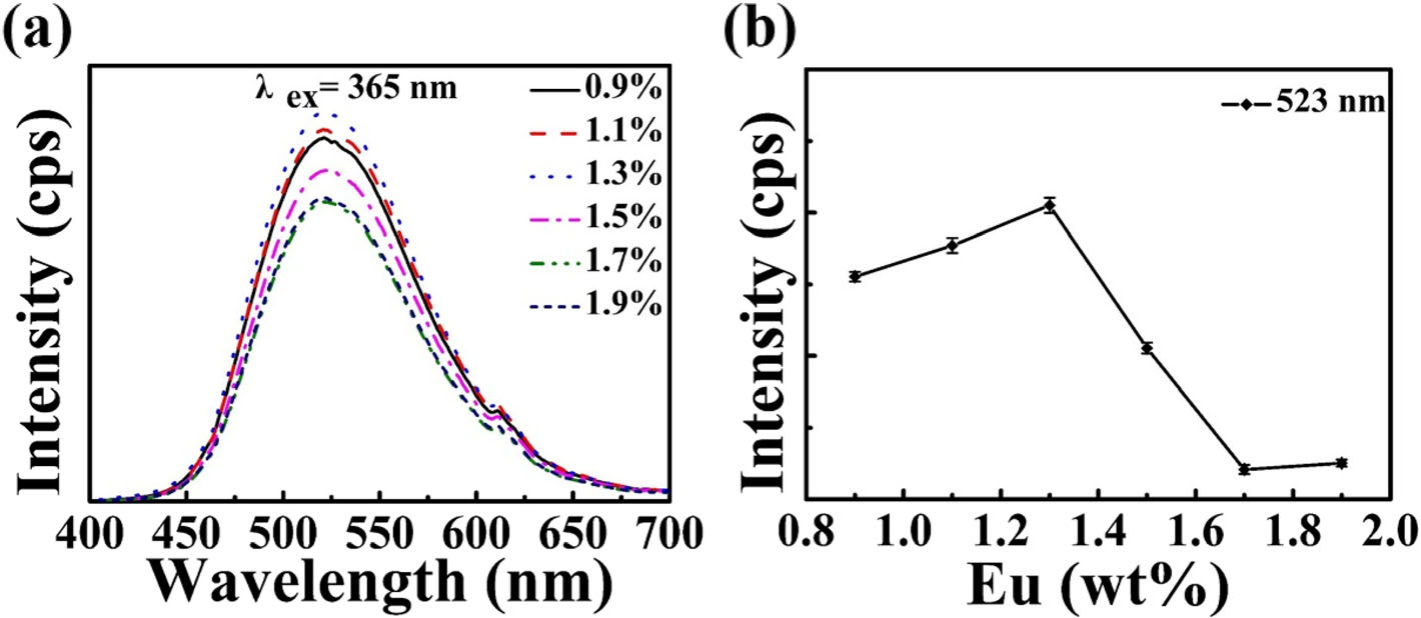
**a** PL emission spectra and **b** the relative PL intensity vary with the concentration of Eu element for zeolite-3A: 1.3 wt% Eu phosphors (*x* = 0.9~1.9) sintered at 1400 °C for 3 h

To optimize the property of zeolite-3A: 1.3 wt% Eu phosphors and observe the effect of Eu element on PL, the PL emission spectra and relative PL intensity for different stoichiometric amounts of zeolite-3A and Eu_2_O_3_ are shown in Fig. 5. It can be seen from Fig. 5a that the emission intensity of Eu^2+^ increases with doping concentration of Eu_2_O_3_ increasing from 0.9 to 1.3%. However, it decreases with the continuous increase of the dopant concentration. It can be observed clearly that the PL effect is the best when the Eu-doping concentration is around 1.3%. It can be explained that the more Eu^2+^ ions, the more luminescent centers. When the concentration of Eu element is beyond 1.3%, the decrease of PL intensity can be attributed to concentration quenching, which is mainly caused by the energy transfer between Eu^2+^ ions. When the concentration of Eu^2+^ ions increase, the distance among Eu^2+^ ions will be short, and then, the energy transfer will increase [42–44]. The error bar curve of relative PL intensity versus Eu-doping concentration is shown in Fig. 5b. It indicates that the change range of relative PL intensity for each Eu concentration is small, meaning that these samples are very repeatable.

**Fig. 6:**
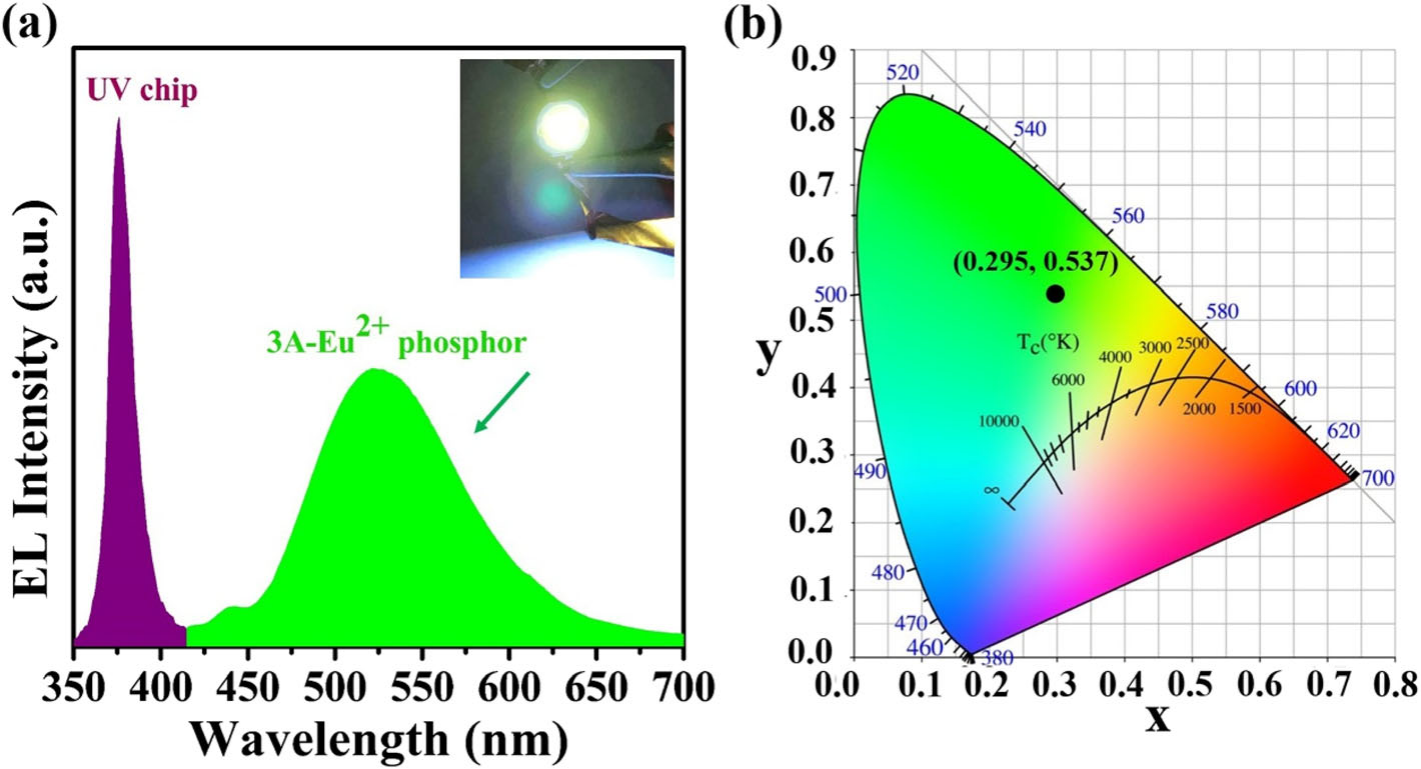
**a** Electroluminescence emission spectrum at 3 V voltage for zeolite-3A: 1.3 wt% Eu phosphor sintered at 1400 °C for 3 h, and the inset is a photograph of working green LED at 3 V voltage. **b** Color coordinate in the CIE1931 diagram

As a proof of lighting application, the green-emitting zeolite-3A: 1.3 wt% Eu phosphor is encapsulated on a UV-emitting chip to fabricate green LED. The EL emission spectrum at 3 V voltage is shown in Fig. 6a. It can be found that the emission peaks of the UV-emitting chip and the green-emitting phosphor are located at ~ 375 nm and ~ 523 nm, respectively. And the inset is the photograph of working green LED that emits bright green light at 3 V voltage. The color coordinates (Fig. 6b) is calculated to be (0.295, 0.537) for the resulting green LED, indicating superior green color purity.

## Conclusions

In this work, we have obtained a bright green emission zeolite-3A: Eu^2+^ phosphor with a quantum yield of about 36.6% and the emission peak located at 523 nm through a green and eco-friendly high-thermal reaction method without any reducing atmosphere. In addition, the sample has a broad excitation band in the range of 310–450 nm, which can be corresponded to the commercial UV-chip excitation (actually, *λ* = 375 nm). Eu^2+^ ions can be gradually incorporated in the zeolite host lattices as the calcination temperature increases. Our research shows that the optimum sintering temperature is 1400 °C and the best doping concentration of Eu ions is 1.3%. Making use of the green-emitting zeolite-3A: Eu^2+^ phosphor encapsulated on a UV-emitting chip, a good green LED with the Commission Internationale de L’Eclairage (CIE) color coordinates at (0.295, 0.537) and brightness of 231.6 cd/m^2^ is obtained. And the green emission zeolite-3A: 1.3 wt% Eu phosphors with increasing luminescent properties will be promising applications for lighting and display.

## Data Availability

The datasets supporting the conclusions of this article are available in the article.

## Abbreviations

CIE: Commission Internationale de L’Eclairage
EL: Electroluminescence
Eu: Europium
FESEM: Field emission scanning electron microscope
LED: Light-emitting diode
PL: Photoluminescence
PLE: Photoluminescence excitation
RE: Rare-earth
TG: Thermogravimetric analysis
XPS: X-ray photoelectron spectroscopy
XRD: X-ray diffraction
